# Health‐Related Quality of Life and Patient‐Centered Care: Measurement Matters

**DOI:** 10.1002/wjs.70099

**Published:** 2025-09-24

**Authors:** Jason B. Liu

**Affiliations:** ^1^ Section of Surgical Endocrinology Department of Surgical Oncology MD Anderson Cancer Center Houston Texas USA; ^2^ Department of Health Services Research MD Anderson Cancer Center Houston Texas USA; ^3^ Patient‐Reported Outcomes, Value, and Experience Center Mass General Brigham Boston Massachusetts USA

Although the twin goals of medicine are to save and improve lives, the latter has often lagged in measurement. Thanks to incredible scientific discoveries, many diseases, including Graves' disease, are now rarely life‐threatening. To keep progressing, modern clinical research must take the measurement of “living better” as seriously as “living longer.”

In this issue, Fung and colleagues are to be congratulated for placing the patient's perspective at the center [1]. They compared patient‐reported outcomes (PROs) after radiofrequency ablation (RFA), radioactive iodine (RAI), and surgery for relapsed or persistent Graves' disease at 2 years. Patients in all three groups were biochemically euthyroid with no active thyroid eye disease. If the study ended here without considering PROs, all three definitive treatments would appear equivalent. However, by pairing a thyroid‐specific patient‐reported outcome measure (PROM), the ThyPRO‐39, with generic (SF‐12) and preference‐based (EQ‐5D‐5L, SF‐6D) PROMs, they deliver decision‐relevant evidence that complements biochemical control and adverse events. On ThyPRO‐39, patients treated with RFA reported significantly lower (better) depressive symptom scores than those treated with surgery or RAI, with numerically favorable differences in anxiety, hypothyroid symptoms, tiredness, overall health‐related quality of life (HRQL) impact, and the composite score. By contrast, SF‐12 composites and utilities (EQ‐5D‐5L, SF‐6D) were similar across groups at 2 years. In clinical terms, a disease‐specific signal favored RFA on mood‐ and energy‐related domains, whereas overall generic health status and preference‐based utility converged. Acknowledging limitations, this study is precisely the kind of PROMs‐forward comparative effectiveness work that can sharpen counseling and support shared decision‐making.

The results intersect meaningfully with contemporary surgical practice. Thyroidectomy remains an essential option for patients with large goiters causing compressive symptoms, suspicious or coexistent nodules requiring pathologic diagnosis, or those seeking immediate definitive control. It also avoids radiation exposure and, in experienced hands, achieves durable biochemical cure in exchange for lifelong thyroxine replacement and uncommon risks for hypoparathyroidism and nerve injury. RAI offers a non‐operative route to definitive therapy, but is not always successful and can also require lifelong thyroid hormone replacement. RFA, an emerging adjunct, preserves some functioning tissue, may obviate routine replacement in many, and, in this study's steady‐state comparison, aligned with better depressive symptom scores. For patients whose priorities include medication independence and improvements in mood and energy, the RFA profile seen here may become a compelling part of the conversation once additional high‐certainty evidence clarifies.

Taking a step away from the clinical implications, what can others seeking to rigorously understand the patient perspective learn from this study? First, choosing the “right” PROM for a research study is not administrivia but the foundation of valid science. Even a beautifully randomized study completely free of bias cannot overcome outcomes that are incorrectly specified. Graves' disease can negatively affect HRQL due to neuropsychological symptoms (e.g., anxiety, low mood, and cognitive complaints), fatigue, and eye discomfort, with broader limitations in daily and social roles. The ThyPRO‐39 directly taps into these domains because the measure was developed from rigorous qualitative research, which forms the fundamental backbone of any high‐quality PROM [2, 3]. It is therefore compelling, but not surprising, that the clearest signal emerged on the ThyPRO‐39. For researchers, the charge is clear: (1) align instruments with the symptom profile patients (not clinicians) say matters most, (2) pre‐specify domain hypotheses, and (3) report both mean changes and responder proportions anchored to minimally important differences. Doing so maximizes responsiveness and reduces the risk that true treatment differences are masked by inadequate measurement rigor.

Second, this study's use of both condition‐specific and generic PROMs is a strength and offers a practical blueprint. Generic measures enable cross‐condition comparisons by providing population‐normed context. However, they can be blunt instruments for the granular symptomatology of certain conditions, such as Graves' disease [4]. Although the current best practice is to combine both condition‐specific and generic PROMs, this approach is not where the future lies. Modern measurement theory, specifically Rasch measurement theory and item response theory (IRT), offer concrete advantages over legacy PROMs based on classical test theory (CTT) [5]. CTT total scores are sample‐dependent and typically ordinal, that is, two patients with the same score may not have the same level of the underlying construct (e.g., fatigue) and change scores can be hard to interpret across subgroups. IRT‐based instruments, in contrast, estimate person scores on an interval‐level metric, yield item and test information functions that show where the scale is most precise, and allow investigators to target items to the severity levels most relevant to the comparison (e.g., the early postoperative period vs. longer‐term steady state). Critically for comparative effectiveness research, IRT facilitates explicit testing for differential item functioning (DIF) across treatment groups; demonstrating invariance increases confidence that differences in scores reflect differences in health, not differences in how the questions were answered after RFA, RAI, and surgery.

Third, the inclusion of health utility indices deserves recognition. Preference‐based measures convert health states into utilities suitable for cost‐utility analyses and value discussions, linking patient experience to policy decisions [6]. In Graves' disease, where trajectories may include transient postoperative disutility, medication burden, or risk of hypothyroidism, utilities help quantify trade‐offs patients care about. As utility data accumulate, several guardrails will further enhance credibility: specify the valuation set relevant to the policy context; consider ceiling effects in milder fluctuating conditions; and, when feasible, report utilities at multiple clinically meaningful time points (e.g., early recovery and steady state) to illuminate transient burdens versus durable gains. That the study focused on a 2‐year steady‐state comparison is a sensible starting point and longitudinal utility trajectories would be a valuable next step. Indeed, as IRT‐based ecosystems mature, utility indices derived from those item banks offer an additional source of valuable information for cost‐effectiveness analyses.

On balance with the study's limitations, this work does what good comparative studies should: it keeps biochemical indices on the table while adding how patients actually feel and function in daily life. For surgeons and researchers, the practical message is clear. PROMs can differentiate treatments even when labs converge; pairing disease‐specific and generic measures is complementary rather than redundant; and integrating preference‐based indices alongside PROMs brings patient experience into the value conversation without blunting important symptom‐level differences. Most importantly, the specific findings observed by Fung et al. (better depressive symptoms after RFA with similar generic health status and utilities across modalities at 2 years) provides a concrete patient‐centered talking point for future consultations and a step toward incorporating PROMs into routine clinical care. As future studies using PROMs thoughtfully gain traction, pre‐registering PROM endpoints, specifying minimally important differences, collecting baseline and longitudinal measures, and ensuring appropriate valuation sets for utilities will make PROM‐anchored comparisons even more compelling for guidelines and shared decision‐making.

By elevating how patients feel and function to the same plane as how their labs look, this study advances a patient‐centered standard for comparing definitive treatments in Graves' disease. It is an important step toward conversations that are informed not just by cure and complications but by the everyday life patients hope to live.

## Author Contributions

**Jason B. Liu:** conceptualization (lead), methodology (lead), project administration (lead), supervision (lead), writing – original draft preparation (lead), writing – review and editing (lead).

## Conflicts of Interest

The author declares no conflicts of interest.
